# Evaluation of the Allplex^TM^ Gastrointestinal Panel—Parasite Assay for Protozoa Detection in Stool Samples: A Retrospective and Prospective Study

**DOI:** 10.3390/microorganisms8040569

**Published:** 2020-04-15

**Authors:** Brice Autier, Jean-Pierre Gangneux, Florence Robert-Gangneux

**Affiliations:** Irset (Institut de Recherche en Santé Environnement Travail), Univ Rennes, CHU Rennes, Inserm, EHESP, UMR_S 1085, 35000 Rennes, France; brice.autier@chu-rennes.fr (B.A.); jean-pierre.gangneux@univ-rennes1.fr (J.-P.G.)

**Keywords:** *Giardia duodenalis*, *Entamoeba histolytica*, *Cryptosporidium* spp., *Dientamoeba fragilis*, *Cyclospora cayetanensis*, *Blastocystis hominis*

## Abstract

This study aims at evaluating the performances of the multiplex PCR Allplex^TM^ Gastrointestinal Panel-Parasite Assay (GIPPA), which detects *G. duodenalis*, *Cryptosporidium* spp., *E. histolytica*, *D. fragilis*, *B. hominis*, and *C. cayetanensis*, by comparison to microscopy. A retrospective evaluation was conducted on a series of positive clinical samples (*n* = 99) stored at −80 °C or at +4 °C. A five-month prospective study was then conducted on all samples sent to our lab for parasite detection (*n* = 586). In the retrospective cohort, sensitivity was 81% for both *G. duodenalis* (26/32) and *D. fragilis* (21/26) and 100% for *Cryptosporidium* spp. (26/26, including 6 different species), *B. hominis* (26/26), and *C. cayetanensis* (4/4). During the prospective study, 95 samples were positive by microscopy and 207 by multiplex PCR assay. The molecular assay showed a significantly higher sensitivity of PCR, especially for *G. duodenalis* (100% vs. 60.7%, *p* < 0.01), *D. fragilis* (97.2% vs. 14.1%, *p* < 0.001), and *B. hominis* (99.4% vs. 44.2%, *p* < 0.001) but also for *E. histolytica* (100% vs. 50.0%). The sensitivity of the Allplex^TM^ GIPPA on the first stool sample was equivalent to the sensitivity of microscopy on multiple stool samples but inferior to multiplex PCR on multiple stool samples. Taken together, the Allplex^TM^ GIPPA is suitable for the routine detection of protozoa in fecal samples.

## 1. Introduction

Infectious diarrheas are among the most life-threatening and invalidating infectious diseases in the world, particularly in children under five years. In 2015, they caused 1.3 million deaths in the world [1]. Diagnosis is sometimes difficult because of the great diversity of pathogens potentially responsible for these intestinal symptoms. For these reasons, and because they are globally less frequent than viral and bacterial infections, parasitic diseases that are due to soil-transmitted helminths and protozoa are often neglected. Yet protozoa represent a major cause of infection, (i) in terms of mortality, with amebiasis and cryptosporidiosis being responsible for respectively 11,000 and 42,000 deaths yearly [2,3], and (ii) in terms of frequency, with pathologies such as giardiasis and dientamoebiasis [4,5]. These protozoa are also very prevalent in high income countries. Microscopic examination of stools remains the reference method for the diagnosis of most intestinal protozoa. This technique however requires three successive samples for the same patient and trained operators and several concentration techniques for optimal results. This approach is time-consuming and yields limited sensitivity. There is, therefore, a need for new methods for the diagnosis of enteric protozoa. Molecular biology—particularly multiplex PCR—seems to offer performances similar to microscopy [6,7] but is limited by the number of parasite species detected.

The recently marketed assay Allplex^TM^ Gastrointestinal Panel-Parasite Assay (GIPPA) (Seegene, Seoul, Korea) is able to detect most protozoa pathogens, i.e., *Giardia duodenalis*, *Cryptosporidium* spp., *Entamoeba histolytica*, *Dientamoeba fragilis*, *Blastocystis hominis*, and *Cyclospora cayetanensis*. In this study, the performances of this assay are evaluated on both retrospective and prospective cohorts.

## 2. Materials and Methods

### 2.1. Clinical Samples

First, 89 clinical samples positive for *G. duodenalis*, *Cryptosporidium* spp., *E. histolytica/dispar/moshkovskii*, *D. fragilis* and/or *B. hominis* by routine microscopic examination were retrospectively analyzed. For each stool analyzed over a three-year period (2015–2018), an aliquot was stored at −80 °C until DNA extraction. This allowed the selection of positive samples for the PCR evaluation. The routine procedure consists of the wet mount examination of fresh stool and various in-house concentration methods based on clinical data (Bailenger’s, Thebault’s, and/or merthiolate-iodine-formalin biphasic methods). *Cryptosporidium* spp. and *Cyclospora cayetanensis* detection relied on Henriksen’s modified Ziehl–Neelsen staining. Finally, four positive samples for *C. cayetanensis* (collected between 2005 and 2009, stored at +4 °C), and 10 *Cryptosporidium*-positive stools provided by the French National Reference Centre for Cryptosporidiosis and identified at species-level were also included. The final retrospective panel contained 103 positive samples including 33 *G. duodenalis*, 15 *E. histolytica/dispar/moshkovskii*, 27 *Cryptosporidium* sp., 26 *D. fragilis*, 27 *B. hominis* and 4 *C. cayetanensis*, possibly associated with other protozoa and helminths.

Secondly, during a five-month period (September 2019–February 2020), all stool samples routinely analyzed in our laboratory were prospectively included for analysis with the Allplex^TM^ assay. The prospective panel consisted of 588 stools from 350 patients.

### 2.2. Multiplex PCR Testing

DNA extraction was performed using the automated device MICROLAB^®^ STARlet (Hamilton Company, Reno, NV, USA) with the STARMag 96 Universal Cartridge kit, following the manufacturer’s instructions. Briefly, a small amount (140–180 mg) of stool was suspended in a Cary-Blair Medium (FecalSwabTM, Copan Diagnostics Inc, Murrieta, CA, USA), vigorously mixed, and, after a 10 min incubation at room temperature, was centrifuged 10 min at 2000 *g* before processing. Extraction was then performed on 50 µL of supernatant and eluted in 100 µL. For amplification with the Allplex^TM^ assay, an internal control DNA (provided) was added to the medium before extraction. The reaction mix and DNA extract were displayed in 96-wells plates by the MICROLAB^®^ STARlet. All PCR runs included both positive and negative controls. Amplification was realized on a CFX96 (Bio-Rad, Marnes-la-Coquette, France) and managed with CFX Manager IVD 1.6 software. Results were analyzed with Seegene Viewer^®^ software. Positive stools for *E. histolytica* detection were confirmed with the G-DiaPara^TM^ assay (Diagenode Diagnostics, Liège, Belgium) following the manufacturer’s instructions.

### 2.3. DNA Preservation in Cary-Blair Suspension

We evaluated whether the FecalSwab^TM^ stool suspensions could be reliably analysed after different conditions of storage. The aim was to assess the possibility of analysing grouped samples. Hence, the differences in signal intensities (expressed in C_T_ values) before and after storage were computed for each stool suspension. Different storage conditions were tested (room temperature and +4 °C) between 0 and 7 days. The samples included in this study were positive for *B. hominis* (*n* = 15), *D. fragilis* (*n* = 9), *G. duodenalis* (*n* = 6), *Cryptosporidium* sp. (*n* = 2), and *E. histolytica* (*n* = 1).

### 2.4. Statistical Analysis

Differences in sensitivities were analyzed with McNemar’s Test. To determine the sensitivity of the assay, true positive and false negative results were determined according to microscopy or to microscopy and PCR assay when specified. The impact of the storage conditions were analyzed through the calculation of differences in cycle threshold (CT) values before storage (C_T_(D_0_)) and after storage (C_T_(D_X_)). This was done for various storage times and temperatures (storage either at 4 °C or at room temperature). For each condition, the median was compared to a hypothetical value of 0 using the Wilcoxon signed-rank test to assess the preservation of the DNA.

## 3. Results

### 3.1. Retrospective Cohort

Four samples presented invalid results (no amplification of an internal control DNA) and were excluded from the subsequent calculations (1 *G. duodenalis*, 1 *E. histolytica/dispar/moshkovskii*, 1 *B. hominis* and 1 *Cryptosporidium* spp.). The final composition of the cohort is available in Table 1.

Sensitivity for the *G. duodenalis* detection was 81% (26/32) (Table 2); the six false negative results were observed for low parasitic loads. The detection of *D. fragilis* had a 81% sensitivity (21/26), with false negative results also related to low parasitic loads. The sensitivity reached 100% for *C. cayetanensis* (4/4), *Cryptosporidium* spp. (26/26), and *B. hominis* (26/26) positive samples. All *Cryptosporidium* species tested (*C. parvum* (*n* = 13), *C. hominis* (*n* = 5), *C. felis* (*n* = 4), *C. canis* (*n* = 1), *C. cuniculus* (*n* = 1), *C. meleagridis* (*n* = 1)) were detected. As the identification at the species level is not possible through microscopy for *E. histolytica/dispar/moshkovskii*, no sensitivity value could be calculated. However, two positive results were obtained and verified with another molecular assay. The 13 other samples that contained *E. histolytica/dispar/moshkovskii* were negative by PCR.

### 3.2. Prospective Cohort

Ninety-five out of 588 samples were positive by microscopy, consisting of 17 *G. duodenalis*, 4 *E. histolytica/dispar/moshkovskii*, 2 *Cryptosporidium* sp., 10 *D. fragilis*, and 72 *B. hominis*. Among them, 10 samples were positive for multiple targets: 6 for *G. duodenalis* and *B. hominis*, 2 for *D. fragilis* and *B. hominis*, 1 for *E. histolytica* and *G. duodenalis*, and 1 for *E. histolytica* and *B. hominis.* With the Allplex^TM^ assay, 207 samples were positive for at least one target: 28 for *G. duodenalis*, 6 for *E. histolytica*, 2 for *Cryptosporidium* sp., 69 for *D. fragilis* and 162 for *B. hominis*. Two samples yielded invalid results (absence of amplification of the internal control) and were excluded from the calculations; both were negative by microscopy and no signal was observed in PCR runs. The sensitivity was 100% (17/17) for *G. duodenalis*, 100% (2/2) for *Cryptosporidium* spp., 98.6% (71/72) for *B. hominis* and 80.0% (8/10) for *D. fragilis,* taking microscopy as the gold standard. During the inclusion period, no *C. cayetanensis* was diagnosed in the laboratory. One sample positive for hematophagous *E. histolytica* and 2 samples positive for *E. histolytica/dispar/moshkovskii* cysts by microscopy were also positive for *E. histolytica* with the Allplex^TM^ assay and confirmed with another PCR assay.

**Figure 1:**
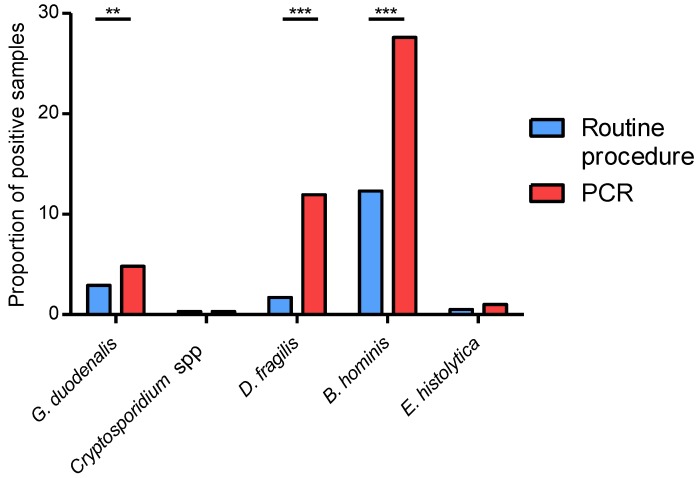
Proportion of positive samples on the prospective cohort (*n* = 586), using routine microscopy and multiplex PCR. **: *p* < 0.01; ***: *p* < 0.001.

Among the samples deemed negative by microscopy, several were positive with the Allplex^TM^ assay greatly increasing the proportion of positive samples compared to routine procedure (Figure 1). This increase was significant for *G. duodenalis* (4.8% vs. 2.9%, *p* < 0.01), *D. fragilis* (11.9% vs. 1.7%, *p* < 0.001), and *B. hominis* (27.6% vs. 12.2%, *p* < 0.001). The difference was not statistically significant for *E. histolytica* detection (1.0% vs. 0.5%), due to the low number of positive samples, but it should be noticed that PCR assay detected two times as many positive samples than microscopy. No additional *Cryptosporidium* sp. was detected with the Allplex^TM^ assay. Finally, the sensitivity was also calculated by combining results of both microscopy and the Allplex^TM^ assay to define positive samples (Table 3). Of note, PCR had a higher sensitivity than microscopy for most pathogens, especially *D. fragilis* (97.2% vs. 13.8%, *p* < 0.001) and *B. hominis* (99.4% vs. 44.2%, *p* < 0.001), but also *G. duodenalis* (100% vs. 60.7%, *p* < 0.01) and *E. histolytica* (100% vs. 50.0%).

### 3.3. Does the Allplex^TM^ GIPPA Need Repeated Samples?

In order to evaluate if clinical laboratories could override the dogma of the multiple stool sampling, the sensitivity of the PCR assay on the first sample was calculated and compared to the sensitivity of the routine procedure on at least three consecutive samples. Only 74 patients had three or more repeated stool samples, and among them 32 had at least one stool positive for *B. hominis*, 13 for *D. fragilis*, 4 for *G. duodenalis,* and none for *E. histolytica* or *Cryptosporidium* sp. The sensitivities of the microscopy on consecutive samples were 78% (25/32), 15% (2/13), and 75% (3/4) for *B. hominis*, *D. fragilis*, and *G. duodenalis*, respectively (Table 4). Sensitivities were either equal or higher with the Allplex^TM^ assay on the first stool, reaching 94% (30/32; *p* < 0.05), 92% (12/13; *p* < 0.01), and 75% (3/4; not significant) for *B. hominis*, *D. fragilis* and *G. duodenalis* respectively.

### 3.4. DNA Preservation in Cary-Blair Medium

**Figure 2:**
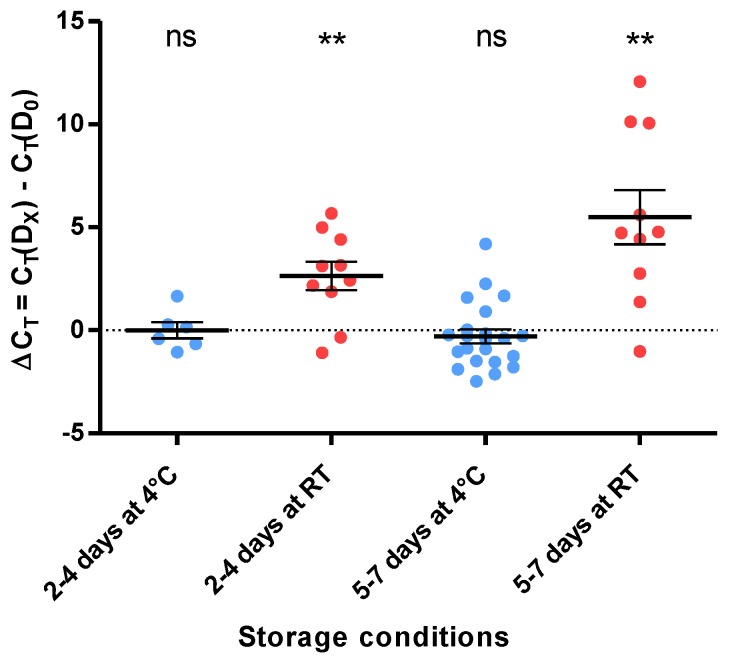
Impact of the storage conditions on the detection signal. Representation of the signal variation in the function of the FecalSwab^TM^ storage conditions. **: *p* < 0.01; ns: not significant; RT: room temperature.

To assess whether the analysis of grouped samples is possible, the impact of different storage conditions of the FecalSwab^TM^ stool suspension on the signal intensity was evaluated. As detailed above, the differences in signal values between the first analysis and after the storage period were calculated (ΔC_T_ = C_T_(D_X_) − C_T_(D_0_)) for different times (2–4 days vs. 5–7 days) and different temperatures (4 °C vs. room temperature). After a storage at 4 °C, the medians of ΔC_T_ were −0.125 and −0.405 for the “2–4 Days” and “5–7 Days” groups, respectively, and were not significantly different from 0 (Figure 2). After storage at room temperature, the medians of ΔC_T_ were 2.780 and 4.750 for the “2–4 Days” and “5–7 Days” groups, respectively, and were in both cases significantly different from 0 by the Wilcoxon signed-rank test (*p* < 0.01).

## 4. Discussion

In the retrospective study, while the sensitivity was excellent for *Cryptosporidium* spp., *C. cayetanensis*, and *B. hominis*, some false negative results were observed for *G. duodenalis* and *D. fragilis*. All *Cryptosporidium* species tested were positive, which is an important point, as many species can infect humans [8]. During the prospective study, sensitivity was excellent for all species, and only rare false negative results were observed for *B. hominis* and *D. fragilis*. No false negative results were observed for *G. duodenalis* in the prospective study. Performances of the Allplex^TM^ GIPPA assay performed on the first stool were equivalent to that of microscopy on multiple consecutive stools, but repeating PCR on consecutive samples yielded even higher sensitivities. Finally, this study assessed that PCR performances were not affected by the storage of the FecalSwab^TM^ stool suspension at 4 °C until 7 days.

The multiplex PCR assay Allplex^TM^ GIPPA showed excellent performances for protozoa detection, even higher than that of other marketed PCR assays such as the BD Max^TM^ Enteric Parasite Panel, G-DiaPara^TM^, or ParaGENIE G-Amoeba assays [9,10,11,12] and with additional targets. Indeed, the sensitivity of these assays ranged from 41% to 96% for *G. duodenalis* [9,10,11], depending on the extraction method and the assay, and the ParaGENIE G-Amoeba assay had 67% sensitivity for *E. histolytica* detection [12]. Moreover, the BD Max^TM^ and G-DiaPara^TM^ assays detected only *C. parvum/hominis*, and none of these detected *C. cayetanensis, D. fragilis*, or *B. hominis*. Few studies evaluated the performances of the Allplex^TM^ GIPPA. Among them, one included a unique stool positive for parasites (*Cryptosporidium* spp.) which was detected by the assay [13]. Another study, with a more consistent cohort, observed performances slightly lower to ours, with 92%, 100%, and 78% sensitivity for *G. duodenalis*, *E. histolytica* \, and *Cryptosporidium* spp., respectively [14]. However, this study was conducted retrospectively, on DNA which had not been extracted with the recommended Hamilton device (MICROLAB^®^ STARlet or NIMBUS).

Regarding the *G. duodenalis* detection, sensitivity was only 81% during the retrospective study, but it should be noticed that the analysis was not performed on fresh stools but on frozen samples without preservative, and this could possibly explain some false negative results [15]. Moreover, the undetected samples contained very low parasite loads and underwent long-time freezing, which could have led to DNA degradation. Interestingly, during the prospective study, PCR showed higher sensitivity than microscopy, which supports this hypothesis. For *D. fragilis* and *B. hominis* few false negative results occurred in retrospectively and prospectively analyzed samples. This appears as surprising, as during the prospective study, the Allplex^TM^ GIPPA assay was significantly more sensitive than the microscopy for these parasites. As an explanation, it could be hypothesized that (i) DNA could have been degraded in retrospectively analyzed samples, as per the *G. duodenalis* example, and (ii) some positive results by microscopy could be a “false positive”. It should indeed be remembered that the *B. hominis* and *D. fragilis* morphological diagnosis is challenging, even for experienced operators, especially in France, where permanent staining (as Wheatley’s trichrome or iron hematoxylin) are not current practice. In the case at hand, almost all but one of the samples that were positive only by microscopy for *B. hominis* and *D. fragilis* contained numerous leukocytes, arthroconidias, or other protozoa which could have led to misdiagnosis.

The numerous samples that were negative by microscopy and positive by PCR raised the question of the specificity of the assay. For *E. histolytica* detection, specificity has been ensured by other molecular assays and clinical data: the positive samples were collected from patients with amoebic abscess or with *E. histolytica* observed in another stool sample. A great number of samples were positive for *B. hominis* only by PCR. These were not verified by other molecular assays but are most likely a true positive of the PCR. Indeed, most of them (71%, 65/91) were confirmed on another stool sample from the same patient, 34% by microscopy (31/91) and 37% by PCR only (34/91). Besides, the relative sensitivity of microscopy was 44%, which is in line with the 47% sensitivity observed in previous works [16]. Likewise, 84% (52/62) of the samples positive by PCR only for *Dientamoeba fragilis* were confirmed on another sample from the patient (16% by microscopy (10/62) and 68% by PCR only (42/62)). In this study, microscopy showed dramatically poor results for *D. fragilis* detection (14% sensitivity). This is in line with data from the literature and explains why PCR is now considered as the reference method for *D. fragilis* diagnosis. Additionally, the detection of *D. fragilis* trophozoites relied only on the direct wet mount of non-fixed stool samples, which is known to be of poor value [4] and it has to be underlined that there is no cyst for this protozoa. Finally, for *G. duodenalis* detection, 82% (9/11) of the samples positive only by PCR were confirmed on another stool sample. Moreover, the two remaining samples were clinically evocative of giardiasis.

The major limit of this study was the poor number of *C. cayetanensis* tested, in line with the low prevalence of this intestinal parasite in France. However, as the oocyst wall of *C. cayetanensis* is similar to that of *Cryptosporidium* spp. [17], the DNA extraction performance should be hypothetically equivalent for both parasites. Further studies are necessary to confirm this. Another limit is that all human-infecting *Cryptosporidium* species could not be tested. Nevertheless, we assessed the most encountered species in human pathology. In France, the six species tested were shown to be involved in 98% of human cases: 54% were due to *C. parvum*, 37% to *C. hominis*, 5% to *C. felis*, 1% to *C. meleagridis*, and 1% to *C. canis* [18].

## 5. Conclusions

To our knowledge, this study is the first evaluation of the Allplex^TM^ GIPPA for protozoa detection, performed on both an exhaustive retrospective cohort and an important prospective cohort. First, this assay combines several advantages, i.e., the ease of use (almost fully automated process), a high number of protozoa detected, and excellent sensitivity results. The use of such a technique could improve routine diagnosis of protozoan infections by clinical laboratories, while being far easier to implement, compared to microscopy. However, a limitation of the technique is that helminths and few other protozoa are not targeted. Finally, we assessed that stool suspension in the Cary-Blair medium was stable until 7 days when stored at +4 °C, allowing the analysis of grouped samples, which is more convenient for most clinical laboratories.

## Figures and Tables

**Table 1:** Number of samples included in the retrospective and prospective cohorts and parasites detected by microscopy.

	Sampling Numbers	
	Retrospective Cohort	Prospective Cohort
**Included samples:**	**99**	**586**
Total number	103	588
Invalid results (excluded for analysis)	4	2
**Parasites detected by microscopy:**	**99**	**95**
*Giardia duodenalis*	32	17
*Cryptosporidium* spp. ^1^	26	2
*C. parvum*	13	Nd ^2^
*C. hominis*	5	Nd ^2^
*C. felis*	4	Nd ^2^
*C. canis*	1	Nd ^2^
*C. cuniculus*	1	Nd ^2^
*C. meleagridis*	1	Nd ^2^
*Dientamoeba fragilis*	26	10
*Blastocystis hominis*	26	72
*Cyclospora cayetanensis*	4	0
*Entamoeba histolytica/dispar/moshkovskii*	14	4
*E. histolytica* (identified by PCR)	2	3

^1^ Molecular identification; ^2^ The 2 *Cryptosporidium* spp. observed in the prospective cohort were not identified at species level.

**Table 2:** Performances of the Allplex^TM^ PCR assay compared to microscopy on the retrospective cohort (*n* = 99).

	Sensitivity% (n/N)
*Giardia duodenalis*	81% (26/32)
*Cryptosporidium* spp.	100% (26/26)
*Dientamoeba fragilis*	81% (21/26)
*Blastocystis hominis*	100% (26/26)
*Cyclospora cayetanensis*	100% (4/4)
*Entamoeba histolytica/dispar/moshkovskii*	nd ^1^

^1^ As microscopy cannot allow the species identification, sensitivity and specificity could not be determined for *E. histolytica* detection. However, two positive results were confirmed by other molecular assay.

**Table 3:** Overall sensitivity of the Allplex^TM^ PCR assay and the routine procedure on the prospective cohort (*n* = 586).

	Sensitivity		
	By Routine Procedure % (n/N)	By PCR % (n/N)	Statistical Significance ^1^
*Giardia duodenalis*	60.7% (17/28)	100% (28/28)	**
*Cryptosporidium* spp.	100% (2/2)	100% (2/2)	ns
*Dientamoeba fragilis*	13.8% (10/72)	97.2% (70/72)	***
*Blastocystis hominis*	44.2% (72/163)	99.4% (162/163)	***
*Cyclospora cayetanensis*	na ^2^	na ^2^	na ^2^
*Entamoeba histolytica*	50% (3/6)	100% (6/6)	ns

^1^ **: *p* < 0.01; ***: *p* < 0.001; ns: not significant; ^2^ no *C. cayetanensis* was diagnosed during the study.

**Table 4:** Sensitivity of the Allplex^TM^ PCR assay on the first patient sample, compared to the routine procedure on multiple consecutive samples (*n* = 74).

	Sensitivity		
	By Routine Procedure on Multiple Consecutive Samples % (n/N)	By PCR on First Sample % (n/N)	Statistical Significance ^1^
*Giardia duodenalis*	75% (3/4)	75% (3/4)	ns
*Dientamoeba fragilis*	15% (2/13)	92% (12/13)	**
*Blastocystis hominis*	78% (25/32)	94% (30/32)	*

^1^ *: *p* < 0.05; **: *p* < 0.01; ns: not significant.
